# Is Evolution of Blind Mole Rats Determined by Climate Oscillations?

**DOI:** 10.1371/journal.pone.0030043

**Published:** 2012-01-09

**Authors:** Yarin Hadid, Attila Németh, Sagi Snir, Tomáš Pavlíček, Gábor Csorba, Miklós Kázmér, Ágnes Major, Sergey Mezhzherin, Mikhail Rusin, Yüksel Coşkun, Eviatar Nevo

**Affiliations:** 1 Department of Evolutionary and Environmental Biology and Institute of Evolution, University of Haifa, Haifa, Israel; 2 Department of Systematic Zoology and Ecology and Department of Paleontology, Eötvös Loránd University, Budapest, Hungary; 3 Department of Zoology, Hungarian Natural History Museum, Budapest, Hungary; 4 Schmalhausen Institute of Zoology, Kiev, Ukraine; 5 Department of Biology, Science and Art Faculty, Dicle University, Diyarbakır, Turkey; University of Western Ontario, Canada

## Abstract

The concept of climate variability facilitating adaptive radiation supported by the “Court Jester” hypothesis is disputed by the “Red Queen” one, but the prevalence of one or the other might be scale-dependent. We report on a detailed, comprehensive phylo-geographic study on the ∼4 kb mtDNA sequence in underground blind mole rats of the family Spalacidae (or subfamily Spalacinae) from the East Mediterranean steppes. Our study aimed at testing the presence of periodicities in branching patterns on a constructed phylogenetic tree and at searching for congruence between branching events, tectonic history and paleoclimates. In contrast to the strong support for the majority of the branching events on the tree, the absence of support in a few instances indicates that network-like evolution could exist in spalacids. In our tree, robust support was given, in concordance with paleontological data, for the separation of spalacids from muroid rodents during the first half of the Miocene when open, grass-dominated habitats were established. Marine barriers formed between Anatolia and the Balkans could have facilitated the separation of the lineage “*Spalax”* from the lineage “*Nannospalax”* and of the clade “*leucodon*” from the clade “*xanthodon*”. The separation of the clade “*ehrenbergi*” occurred during the late stages of the tectonically induced uplift of the Anatolian high plateaus and mountains, whereas the separation of the clade “*vasvarii*” took place when the rapidly uplifting Taurus mountain range prevented the Mediterranean rainfalls from reaching the Central Anatolian Plateau. The separation of *Spalax antiquus* and *S. graecus* occurred when the southeastern Carpathians were uplifted. Despite the role played by tectonic events, branching events that show periodicity corresponding to 400-kyr and 100-kyr eccentricity bands illuminate the important role of orbital fluctuations on adaptive radiation in spalacids. At the given scale, our results supports the “Court Jester” hypothesis over the “Red Queen” one.

## Introduction

**Figure 1:**
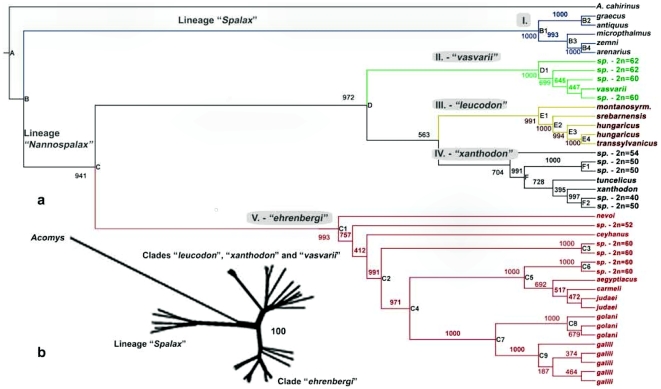
Maximum likelihood phylogenetic tree showing the relationship between 41 samples of blind mole rats plus one outgroup *Acomys cahirinus*. Numerical values represent bootstrap values out of 1000 reiterations. The nodes marked by capital letters represent internal and external branches supported by bootstrap values higher than 95%. The clades are referred to by Roman numerals: I “Spalax”, II “vasvarii”, III “leucodon”, IV “xanthodon” and V “ehrenbergi” (a). Taxonomic nomenclature is according to Table S1; Neighbour-net graph showing an agreement in the major splits with the ML tree (b).

**Figure 2:**
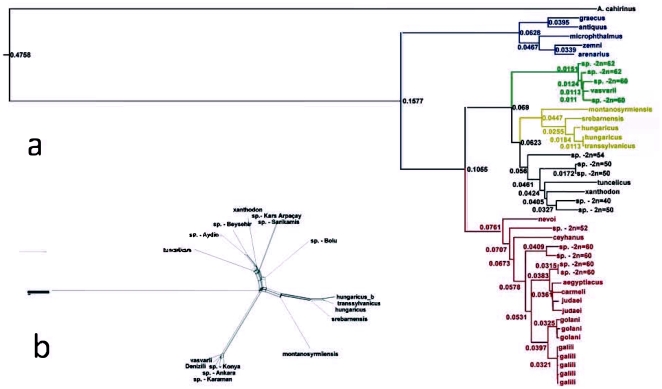
Maximum likelihood phylogram of blind mole rates constructed by means of PhyMl. Branch values indicate the rate of heterogeneity (a). Neighbor-net graph showing relationship between clades “*vasvarii”,* “*leucodon”* and “*xanthodon”*. It shows that the clades are significantly separated (indicated by 100% bootstrap), but that among them some speciation-related processes such as hybridization or introgression could take place (b).

**Figure 3:**
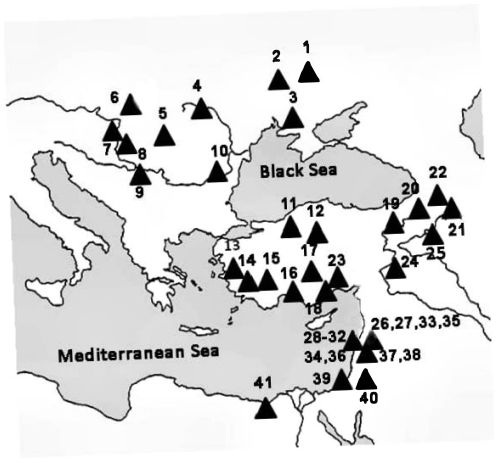
Sampling points covering the distribution area of blind mole rats ranging from the Carpathian Basin to the East Mediterranean. For numerical symbols see Table S1.

It has been suggested that the “Court Jester” model [1] which, recognizes the important role of climate change on speciation, and the “Red Queen” model which, promotes biotic over abiotic interactions [2], describe evolution at different time scales [3]. The paleobiological studies indicate that short time scale evolution concerns biotic interactions in ecosystems such as competition, predation, and cooperation, but that large-time scale patterns of biodiversity are driven by the physical environment, including geological and tectonic events, landscape, food supply, or climate [3]. The application of a phylogenetic approach [4] on the molecular evolution of clades offers deep insight into the role of intrinsic and extrinsic factors on the evolution of blind mole rats (namely, spalacids in the following text). If conflicting signals are present, combinations of bifurcating-based and network-split phylogenetics methods are required [5], [6]. Fossorial spalacids, as all subterranean mammals, display convergent molecular and organismal adaptations to life underground [7]. They have been traditionally poor in number of taxa [8]. Currently, only 13 species were listed in the last edition of Mammal Species of the World [9]. However, based on chromosome analyses, a larger number of species should be expected [7]. Some authors regard *Nannospalax* as a junior synonym of *Spalax* [9], but monophylogenic analysis of the group has not been performed so far. A majority of spalacid species is living mostly in the Middle East steppes whose contraction and expansion dynamics have been controlled by climate change, including Paleoclimate periodicities [10], and by frequent geological and tectonic events. Apart from landscape fluctuations resulting from geological and tectonic events, we expect that the paleoclimate periodicities could also be conducive to adaptive radiation in spalacids. The climate changes would influence spalacids, living in the underground of steppes, mostly indirectly by affecting vegetation that acts as a population filter [7]. In other words, we expect to find that the landscape fluctuations, and the palaeoclimate periodicities and shifts leading to the origin of steppes and/or steppe isolates correspond to branching events in the molecular phylogenetic tree (Figures 1, 2) constructed from samples covering a large part of the blind mole rats distribution (Figure 3). In spite of the inadequacy of our current methods to capture the complex nature of mtDNA evolution over large time scales, the inferred age estimates support the paleontological data, tectonic and geological events, and the frequency of periodic palaeoclimate changes. At the given scale of resolution, this work supports therfore, the “Court Jester” model [1] over the “Red Queen” one [2].

## Results

**Figure 4:**
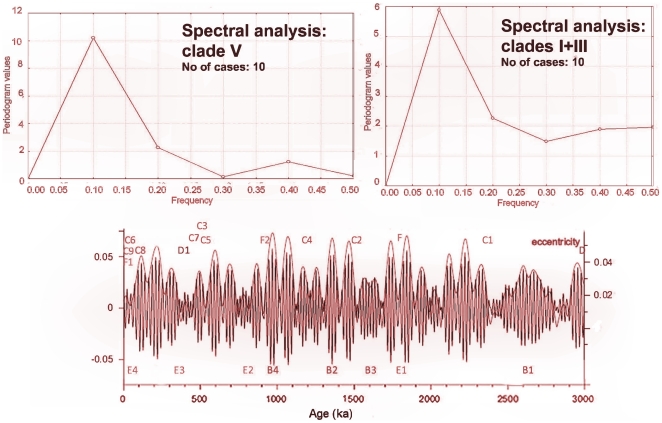
Periodogram of branching events in the clades “*ehrenbergi*” (a) and “*leucodon*” (b). Frequency a = 0.1 is significant (*P*<0.05). Periodicity in eccentricity during the last 3 My with marked branching events in blind mole rats.

The novel sequences were deposited in the GeneBank database under the accession numbers HQ652108 to HQ652327. Since the ML phylogenetic trees constructed for each mitochondrial partial sequence were congruent, only the tree built from the combined data was used in the consecutive analyses. The tree is composed of 41 samples of the spalacids and one outgroup *Acomys cahirinus*. The samples of spalacids represent either biological or chromosomal species with the exception of *galili, golani, judaei* and sp. 2n = 60, in which specimens representing different populations, but not different species, might be involved. Significant cases of homologous recombinations were not detected in the data set. The Akaike Information Criterion (included in Hyphy Package: [11]) identified the optimal substitution models GTR+G+I ( = REV model). This general time-reversible model allows unequal base frequencies and a unique probability for each of the six possible transformations [12]. Since the global molecular clock was significantly rejected and heterogeneous substitution rates were present along the tree (LR statistic: 69.2227, Degrees of freedom: 1, P = 5.55112e-017), we adapted a relaxed uncorrelated lognormal clock (BEAST) and employed, as an external calibration point for molecular dating, the earliest fossil record of a split between “*Spalax*” and “*Nannospalax*” (mean = 7.67 Ma, st. dev. = 0.57). This implies that the dating of the separation between “*Spalax*” and “*Nannospalax*” is rather imposed on the tree than inferred from it. The Maximum Likelihood (ML) tree has most of its branches well supported by bootstrap values at the level of lineages and clades. The only exception is the split between clade III “*leucodon*” and clade IV “*xanthodon*”. The ML tree is in consensus with the neighbor-net graph regarding major splits (Figure 1). However, the neighbor-net graph better illustrates the split between the “*Acomys*” outgroup and the “*Spalax*” lineage. In spite of the generally good support for the branching events on the ML tree, and in spite of all the splits in the lineage “*Spalax*” and the clade “*leucodon*”, there are instances of poor resolutions in the clades “*xanthodon*”, “*ehrenbergi*” and “*vasvarii*” that may indicate that spalacids evolution did not proceed in a strictly bifurcating way. From the neighbor-net graph (Figure 4) on the clades “*vasvarii*”, “*leucodon*” and “*xanthodon*”, it becomes immediately visible that the spalacids evolution and radiation have a more complex history than previously assumed, at least in this part of the tree.

Partial autocorrelation of all branching events in spalacids (Table 1) showed a significant ∼400-kyr periodicity (N = 25, ACF(k) = 0.39, T-STAT = 1.95, *P* = 0.03). In the clade V. “*ehrenbergi*”, we identified a significant 100-kyr periodicity (Figure 4, Fourier transform, N = 10, α = 0.1, cosine = 1.228, sine = −0.729, *P*<0.05) like in the clades I+III from the Balkans (Figure 4, Fourier transform, N = 10, α = 0.1, cosine = 0.757, sine = −0.77, *P*<0.05). However, we observed a shift in the amplitude of periodicity between both groups (Bivariate Fourier Analysis, N = 10, α = 0.1, cosine (I+III) = 0.757, sine (I+III) = −0.77, cosine (V) = −0.05 and sine (V) = 0.154) as well as in the clades (II+IV) from Anatolia (a sample size too small for a detailed analysis).

**Table 1:** Timing of branching events on “significant” nodes (bootstrap>95) on the Maximum likelihood tree (Figure 1).

Node	Mean	Interval (95%)	Node	Mean	Interval (95%)
A	19.9531	[12.8489–27.4378]	C7	0.5191	[0.3068–0.7328]
B	7.5651	[6.4697–8.7122]	C8	0.1461	[0.0706–0.225]
B1	2.6704	[1.8574–3.4955]	C9	0.0596	[0.0258–0.0957]
B2	1.3621	[0.8076–1.9249]	D	3.1132	[2.2517–3.9619]
B3	1.6372	[1.1114–2.2343]	D1	0.403	[0.2547–0.5705]
B4	0.9794	[0.6006–1.3742]	E1	1.81	[1.1933–2.3856]
C	4.6802	[3.4944–5.8574]	E2	0.7569	[0.4725–1.0847]
C1	2.3488	[1.7264–3.12]	E3	0.3684	[0.2006–0.5535]
C2	1.4852	[1.0657–1.9921]	E4	0.0458	[0.0121–0.0896]
C3	0.5153	[0.29–0.7944]	F	1.7751	[1.2558–2.3223]
C4	1.2263	[0.8444–1.6299]	F1	0.0662	[0.0206–0.1209]
C5	0.4875	[0.3021–0.6641]	F2	0.9523	[0.5896–1.3693]
C6	0.0244	[0.0015–0.054]			

## Discussion

The new data and their geographic coverage enabled more detailed analyses of the molecular evolution of spalacids than the earlier attempts [7], [13]–[20]. The solid resolution of the ML phylogenetic tree topology and congruence in major splits between the tree and neighbor-net, as well as the correspondence of molecular dating to paleontological evidence, alleviate concerns about reconstruction artifacts caused by limited taxon sampling and sequence length. Nevertheless, the lack of resolution in some splits indicates the presence of speciation-related processes, such as incomplete lineage sorting and hybridization, in the spalacid evolution. If this is the case, the evolutionary process is network-like based and, consequently, the single phylogenetic bifurcating tree does not describe correctly the situation in which different parts of the data underwent different evolutionary histories [21]. Critically important for the estimation of accurate divergence times is the incorporation of the proper age of calibration [22]. As an external calibration point for molecular dating, we chose the separation between “*Spalax”* and “*Nannospalax”* because of its nearly central position on the constructed phylogenetic tree and of the agreement on this point in our data as well as among most paleontologists and molecular biologists. The existing dating discrepancy between the the earliest known fossils of “*Spalax”* (7.1−5.3 Ma) [23] and of “*Nannospalax”* (8.24−7.1 Ma) [24], [25] was solved by choosing only the dating of “*Nannospalax*, whice indicates a split deeper in the past [26]. The rather broadly estimated time intervals indicate a need to improve our current methods if we want to capture the complex nature of mtDNA evolution in different time scales. Nevertheless, our data allowed the disclosure of hidden periodicity in the molecular data corresponding to the frequencies of the worldwide-shared orbital eccentricity [27]. Orbital eccentricity became the dominant force during the Middle and Late Pleistocene [28], but already certain parts of the Oligocene showed a strong climate response linearly related to variations in the eccentricity band (at 400- and 100-kyr periods) [29]. In the area of the Saharo-Syrian desert belt (bordering with steppes in the southern part of the distribution area of spalacids) increased dust production during eccentricity minima — strongly modulated by the 400-kyr eccentricity cycle and also by the 100-kyr cycle [10] — is associated with precession maxima of increased aridity and reduced vegetation cover as well as with the expansion of the desert biome. The rapid [30] expansion of the desert could have led to the formation of steppe isolates surrounded by a desert inhospitable for mole rats. It is expected that, speciation is easier in small peripheral populations of spalacids than in larger populations [7]. The newly established species could spread during the next part of the climate cycle characterized by the expansion of steppes and the contraction of desert areas. However, the expansion of steppes should not have initiated the speciation since desert isolates are inhospitable to spalacids. Similarly, the expansion of the boreal forest biome during eccentricity maxima could have led to the establishment of steppe isolates surrounded by forests in the northern part of the spalacids geographic distribution range (clades I+III) [31]. The occurrences of speciation in the southern part of the spalacids distribution area during inter-pluvial periods and in the northern part of distribution during pluvial periods should not have influenced the periodicity frequency but instead, as we have shown, produced a shift in the speciation amplitude. periodicities associated with the speciation of spalacids might correspond to the sequences of high and low rates of speciation observed in rodents [8]. The fact that the orbital eccentricity became the dominant force during the Middle and Late Pleistocene [28] could explain why spalacids and some other underground taxa, such as Bathyergidae, Geomyidae, Octodontidae, Myospalacinae, reached or maintained their peaks of diversity from the Pleistocene to the present time [8], and why neither a dramatic cooling during the Oligocene nor the expansion of open grassland habitats during the middle Miocene are correlated with increased originations and diversification of the subterranean mammal clades [8].

### Synthesis: Inferred evolutionary history of spalacids

The obtained tree topology and its chronology, in combination with zoogeographic, geological, tectonic, and palaeoclimate evidence, introduce a testable scenario of the evolutionary history of spalacids.

#### Spalacids/rodent split

Clearly shown is the separation of the outgroup (*Acomys*), representing rodents, from the oldest detected “*Spalax*” lineage in the first half of the Miocene (∼20 Ma; range: 12.8–27.4). The corresponding species of the lineage “*Spalax*” are known only from the Balkans and are missing in Asia Minor, Anatolia, the Levant, and Egypt where only the species representing the lineage “*Nannospalax*” are present. However, “*Spalax*” coexists with “*Nannospalax*” in the Balkans. There is a deep evolutionary separation in the lineage “*Spalax*” into the two clades represented by *Spalax antiquus* within the Carpathians and *S. graecus* outside the Carpathians. The estimated timing of the spalacids/muroid split is overlapping with a period of drier (and warmer or cooler) climate than today and was accompanied by a “transgression” of grasslands [32] and the appearance of open-space adapted mammals [33]. The average of the range corresponds to the dating of earliest known fossils of blind mole rats at about ∼20–24 Ma [34], [35] and to dated splits estimated from DNA-DNA hybridizations (14.7–23.4 Ma [14]), MHC nuclear genes (±20 Ma [36]) and in multiple nuclear genes (19.8 Ma [37]). However, the chaotic and unbalanced taxonomy of mole rats [9] might be a source of confusion, as, for example, the inclusion of Rhyzomyinae into Spalacidae (Spalacinae) changes both molecular and paleontological datings [37], [35]. Estimates of the spalacids/rodent split at 35–48 Ma [38], [39] could result from ignoring heterogeneity of the molecular rate [22].

#### *Spalax/Nannospalax* split

The separation of the lineage “*Spalax”* from the lineage “*Nannospalax”* during the dry late Miocene [40] (∼7.6 Ma; range: 6.5–8.7) could be attributed to the establishment of a marine barrier between Anatolia and the Balkans during the Tortonian (11.6−7.2 My) [41]. These two lineages “*Spalax*” and “*Nannospalax*”, correspond to genera, as proposed by some authors [42] but disputed by others [7]. The few fossil localities [24], [23], we are aware of, are in close proximity to the marine barrier and provided the earliest known fossils of “*Spalax”* (7.1−5.3 Ma) [23] and “*Nannospalax”* (8.24−7.1 Ma) [24], [25]. The present-day area of “*Spalax”* in the western part of its distribution range has been progressively reduced due to pressure from “*Nannospalax”*. In the eastern part, “*Spalax”* has been protected from the “*Nannospalax”* pressure by the high mountain range of the Caucasus (various data for initial uplift of the Caucasus are available from Sarmatian/10 My [43], [44] till Quaternary/2 My [44]).

#### Splits of “*ehrenbergi*”

The separation of clade V. “*ehrenbergi*” (∼4.7 Ma; range: 3.5–5.9) from clades II–III–IV (including clade “*leucodon*”) was suggested earlier, on the basis of rRNA and mtDNA analyses [45]. Today, clade V. “*ehrenbergi*” is today occupying the semi-desert area south of the East Anatolian mountain range. The separation occurred during a tectonically-induced uplift of this range in the late Miocene-early Pliocene era[46]. The 1,500 m isoline clearly separates the lowland and highland areas with “*ehrenbergi”* and “*leucodon”*, respectively. The separation process was probably reinforced when lowland areas became drier during the Mediterranean salinity crisis (5.3–5.8 My) [33].

#### *Vasvarii/leucodon* split

The separation of the clade II “*vasvarii*” from the “*leucodon*” branch (∼3.1 Ma; range: 2.3–4.0) had not been recognized in earlier studies. This separation happened when the rapidly uplifting Taurus mountain range prevented the Mediterranean rainfall from reaching the Central Anatolian plateau. The 1-km high plateau (corresponding exactly to the “*vasvarii*” area) is part of the high Anatolian range since its uplift in the late Miocene-early Pliocene. It has a warm temperate climate, including dry seasons [47]. A separate biogeographic province was formed when the uplifting Taurus Mountains, now 2-km high, prevented the Mediterranean rains from reaching the plateau and produced an arid environment in the rain shadow [48]. The dominance of fluvial deposits from the Pliocene to the present time indicates that this was not due to the shifting of climatic zones, but to the Taurus uplift, dated as 8 My to the present, with intermittent subsidence and renewed uplift during the Quaternary [49], [50]. The aridification induced by the relief change overprinted the slow, middle Miocene-Pliocene global increase of C4 grasses [51].

#### Xanthodon/leucodon split

The divergence (weakly supported by bootstrap: Figure 1) from the common ancestor of clade IV “*xanthodon*” and clade III “*leucodon*” occurred ∼2.7 (range: 1.9–3.4) MYA. The Bosphorus, the Sea of Marmara and the Dardanelles form the barrier between them. There was certainly a marine connection between the Black Sea and the Mediterranean Sea during the Messinian Lago Mare [41]. A short time later, this connection disappeared [41], allowing the migration of “*leucodon”* between Anatolia and the Balkans during the Pliocene. During the latest Pliocene or early Pleistocene, this connection was severed, never to be restored again. Marine straits or major rivers – during the ice ages – separated the Balkans from Anatolia, effectively barring any faunal exchange [52] between “*xanthodon*” and “*leucodon*”. During sea level low-stands in high glacial times, extensive shelves existed in the Aegean region, allowing “*xanthodon*” to populate eastern Aegean islands [53]. Meanwhile, there was a significant outflow from the Black Sea, via the Marmara, to the Aegean Sea [54], effectively maintaining the separation of “*leucodon”* in the Balkans from “*xanthodon*” in Anatolia.

#### *Spalax antiquus/S. graecus* divergence

There is a deep evolutionary separation in the lineage “*Spalax*” into the two clades represented by *Spalax antiquus* within the Carpathians and *S. graecus* outside the Carpathians. The separation of *Spalax antiquus* from *S. graecus* (∼1.3 Ma; range: 0.81–1.9) occurred when the southeastern Carpathians were uplifted. The mountain chain reached about 1-km elevation at 2.5 Ma. A further uplift yielded a 2-km elevation less than 1 Ma [55]. Subsequent erosion caused the present maximum elevation at 1,700 m, but the mountain passes are lower than 1 km above sea level.

## Materials and Methods

The phylogenetic analysis of five mtDNA sequences (12S rRNA, tRNA-Val, 16S rRNA, tRNA-Leu (UUR), NADH1, tRNA-Ile, 3742 bp in total: Table 2) was conducted on 41 samples from 35 different populations of blind mole rats (Table S1) and one outgroup [(*Acomys cahirinus* (Desmarest) collected at Mt. Carmel, Israel)] (Figure 3). After DNA extraction [19] from tissue samples (Table 2) and amplification [19] by selected primers (the list of primers is available from the authors upon request), the obtained and cleaned PCR products were sequenced on ABI PRISM 3130 Genetic Analyzer (Applied Biosystems) following the standard protocol [19]. The samples were independently amplified to avoid producing recombinations in vitro during the polymerase chain reaction (PCR) [40]. After alignment of the data by means of Mega4 [41] that contains the alignment program ClustalW [42], maximum likelihood (ML) phylogenetic trees were constructed by means of PhyML_3 (http://www.atgc-montpellier.fr/phyml) and BEAST [43], respectively. The global molecular clock hypothesis and optimal substitution model were tested or identified with the help of Hyphy [11]. To check for the reliability of the obtained ML tree topology and to solve occurring data conflicts, the sequences were also analyzed by the SplitsTree4 program[5], using the neighbor-net method from uncorrected distance P and by employing the Convex Hull splits transformations [56]. We used a combination of the bifurcating tree-based and network-based approaches [57] because even in the absence of detection of any no significant event, such as the presence of homologous recombinations potentially rendering the phylogenetic approach unreliable [6], [58], this does not mean that this kind of event did not occur. The network-based approach, better suited for description of evolutionary history in the case of data conflicts, has also been used to describe evolutionary history in cases of the mentioned data conflict on the bifurcating tree [5]. The presence of recombinations in the data set was tested by means of 14 relatively powerful tests available in RDP3 [44]. The test of the validity of the molecular clock and the identification of the optimal substitution model were done in Hyphy [11]. As a criterion to choose the optimal model, we used the Akaike Information Criterion [46] that, as was shown, is better performing than commonly employed likelihood test [59]. As an external calibration point, we opted for molecular dating of the separation between “*Spalax”* and “*Nannospalax”* because of the separation position on the constructed phylogenetic tree (neither at its extreme beginning nor at its end), and the general agreement on this point among paleontologists[23], [25]. The external calibration point (mean = 7.67 Ma, st. dev. = 0.57) was estimated from the dating of the earliest known fossils of “*Nannospalax”* (8.24−7.1 Ma) [14], [15]. We did not use the earliest known fossils of “*Spalax”* (7.1−5.3 Ma) [13] assuming that the observed gap between both taxa results from missing early fossils of *Spalax* [26]. The relatively large standard deviation compensates for the dating uncertainty but also contributes to a rather wide range of estimates. Expecting that the estimated absolute dating of branching splits on the phylogenetic tree represents a natural one-way ordering in time, we employed the autoregressive-moving-average (ARMA) model that attempts to understand the values in a data series and perhaps predicts future values. Mainly partial autocorrelation [47] was used to examine the serial dependence of the values and, harmonic analyses (Fourier and Bivariate Fourier Analyses) that look at frequency shifting and modulation properties in discrete time series (included in Statistica for Windows, Ver. 6 (Statsoft Inc.).

**Table 2:** Analyzed mtDNA sequences and their basic parameters.

MtDNA sequence	Position	b.p.	Nvs.	Ns.	Ncs.	Mean			
						T	C	A	G
12S rRNA	68–1029	968	212	43	692	23.8	20.9	37.4	17.9
tRNA-Val	1030–1095	76	16	2	51	20.3	29.7	31.6	18.3
16S rRNA	1096–2670	1603	450	87	1153	24.2	20.5	38.6	16.6
tRNA-Leu	2671–2754	75	17	2	58	26.4	21.2	33.2	19.2
NADH 1 (partial)	2755–3700	946	375	61	570	27.3	28.1	33.2	11.3

Nvs. - Number of variable sites; Ns. - Number of singletons; Ncs. - Number of conserved sites. Base frequencies are A = 0.3654, C = 0.2273, G = 0.1586, T = 0.2488.

## Supporting Information

Table S1 **Analyzed specimens of blind mole rats and their specifications.**
(DOC)
