# Supplementary material for: Is Evolution of Blind Mole Rats Determined by Climate Oscillations?
Source: PLoS One. 2012 Jan 9;7(1):e30043. doi: 10.1371/journal.pone.0030043 (PMC3253805; doi:10.1371/journal.pone.0030043)
Supplement: Table S1 — Analyzed specimens of blind mole rats and their specifications. (DOC) [file pone.0030043.s001.doc]

**Table S1. Analyzed specimens of blind mole rats and their specifications**

| No/Code/Deposit | Taxon | 2n | Locality |
| --- | --- | --- | --- |
| 1/8788/HNHM | *Spalax microphthalmus* Güldenstaedt | 60 | Novomoszkovsz, UA |
| 2/8586/HNHM | *S. zemni* Erxleben | 62 | Krivij Rig, UA |
| 3/8182/HNHM | *S. arenarius* Reshetnik | 62 | Kherson,UA |
| 4/72/HNHM | *S. graecus* Nehring | 62 | Iasi, RO |
| 5/65/HNHM | *S. antiquus* Méhely | 62 | Aiton, RO |
| 6/13/HNHM | **S. transsylvanicus* Méhely | 50 | Józsa, HU |
| 7/68/HNHM | **S. hungaricus* Nehring | 48 | Mezőtúr, HU |
| 8/70/HNHM | **S. montanosyrmiensis* Savić & Soldatović | 54 | Suboticka Pescara, SB |
| 9/3335/HNHM | **S. hungaricus* Nehring | 48 | Susara, SB |
| 10/97/HNHM | **S. srebarnensis* Savić & Soldatović | 48 | Lipnita, RO |
| 11/B01/IE | **S. sp.* | 54 | Bolu, TU |
| 12/AK/IE | **S. sp.* | 62 | Ankara, TU |
| 13/IZ/IE | **S. xanthodon* Nordmann | 38 | İzmir, TU |
| 14/AD/IE | **S. sp.* | 50 | Aydın, TU |
| 15/DZ/IE | **S. sp.* | 60 | Denizili, TU |
| 16/BY/IE | **S. sp.* | 40 | Beysehir, TU |
| 17/KO/IE | **S. sp.* | 62 | Konya, TU |
| 18/KM/IE | **S. sp.* | 60 | Karaman, TU |
| 19/MA/IE | **S. vasvarii* Szunyoghy | 60 | Malatya, TU |
| 20/BN/IE | **S. tuncelicus* Coşkun | 54 | Bingol, TU |
| 21/SR/IE | **S. sp.* | 50 | Sarikamiş, TU |
| 22/103/HNHM | **S. sp.* | 50 | Kars Arpaçay, TU |
| 23/TR/IE | **S. ceyhanus* Szunyoghy | 56 | Tarsus, TU |
| 24/GZ/IE | **S. nevoi* Coşkun | 58 | Gaziantep, TU |
| 25/104/HNHM | **S. sp.* | 52 | Bismil, TU |
| 26/2535/IE | **S. golani* Nevo et al. | 54 | Mt. Hermon, IL |
| 27/623/IE | **S. golani* Nevo et al. | 54 | Quenetra, IL |
| 28/2094/IE | **S. galili* Nevo et al. | 52 | KBZ, IL |
| 29/2095/IE | **S. galili* Nevo et al. | 52 | Alma, IL |
| 30/1067/IE | **S. galili* Nevo et al. | 52 | Alma, IL |
| 31/575/IE | **S. galili* Nevo et al. | 52 | KBZ, IL |
| 32/1034/IE | **S. galili* Nevo et al. | 52 | Alma, IL |
| 33/5121/IE | **S. golani* Nevo et al. | 54 | El Al, IL |
| 34/1404/IE | **S. carmeli* Nevo et al. | 58 | Muhraka, IL |
| 35/185/IE | **S. sp.* | 60 | Irbid, JO |
| 36/Anza/IE | **S. judaei* Nevo et al. | 60 | Anza, IL |
| 37/183/IE | **S. sp.* | 60 | Zarqa, JO |
| 38/180/IE | **S. sp.* | 60 | Madaba, JO |
| 39/Lahav/IE | **S. judaei* Nevo et al. | 60 | Lahav, IL |
| 40/214/IE | **S. sp.* | 60 | Wadi Musa, JO |
| 41/14/IE | **S. aegyptiacus* Nehring | 60 | El Alamein, Egypt |

**S* - genus *Nannospalax* in two genera system, HNHM - Hungarian Natural History Museum, HU - Hungary, IE - Institute of Evolution, University of Haifa, IL - Israel, JO - Jordan, RO -Romania, SB - Serbia, TU - Turkey, UA - Ukraine.
